# Alpha-lipoic Acid suppresses P2X receptor activities and visceral hypersensitivity to colorectal distention in diabetic rats

**DOI:** 10.1038/s41598-017-04283-7

**Published:** 2017-06-28

**Authors:** Ji Hu, Xin Qin, Zhen-Yuan Song, Pan-Pan Yang, Yu Feng, Qian Sun, Guang-Yin Xu, Hong-Hong Zhang

**Affiliations:** 1 Department of Endocrinology, the Second Affiliated Hospital of Soochow University, Suzhou, 215000 P.R. China; 2 Department of Endocrinology, Suzhou Science and Technology Town Hospital, Suzhou, 215000 P.R. China; 3 Department of Endocrinology, the East District of Suzhou Municipal Hospital, Suzhou, 215000 P.R. China; 4 Jiangsu Key Laboratory of Translational Research and Therapy for Neuro-Psycho-Diseases, Institute of Neuroscience, Soochow University, Suzhou, 215123 P. R. China

## Abstract

The present study was designed to investigate the roles of P2X3 receptors in dorsal root ganglion (DRG) neurons in colonic hypersensitivity and the effects of alpha-lipoic acid (ALA) on P2X3 receptor activity and colonic hypersensitivity of diabetic rats. Streptozotocin (STZ) was used to induce diabetic model. Abdominal withdrawal reflex (AWR) responding to colorectal distention (CRD) was recorded as colonic sensitivity. ATP-induced current density of colon-specific DRG (T13-L2 DRGs) neurons was measured with whole-cell patch clamp. The expression of P2X3Rs of T13-L2 DRGs was measured by western blot analysis. The results showed that AWR scores significantly increased after STZ injection. P2X3R expression and ATP current density of T13-L2 DRG neurons were enhanced in diabetic rats. Intraperitoneal injection with ALA once a day for 1 week remarkably reduced P2X3R expression and ATP current density in diabetic rats. Importantly, ALA treatment attenuated colonic hypersensitivity in diabetic rats. Our data suggest that STZ injection increases expression and function of P2X3 receptors of colon-specific DRG neurons, thus contributing to colonic hypersensitivity in diabetic rats. Administration of ALA attenuates diabetic colonic hypersensitivity, which is most likely mediated by suppressing expression and function of P2X3 receptors in DRGs of diabetic rats.

## Introduction

Gastrointestinal dysfunction is one of the chronic complications of diabetes mellitus. The prevalence of gastrointestinal symptoms in patients with diabetes is inconsistent, because of the variations in methodology, patient populations, and the adequacy of the control groups used^1–4^. However, the frequency of symptoms is increased, including nausea and vomiting, abdominal pain, constipation, and diarrhea^5^, which impact adversely on quality of life in patients with diabetes. Therefore, clinicians and researchers have paid more and more attention to gastrointestinal dysfunction of diabetes. However, the underlying pathophysiology of these conditions remains unclear.

Previous studies suggest that diabetes-induced sensory neuropathies may be the most important factor in visceral hypersensitivity. Diabetes resulted in increased activity of primary afferent fibers and transmitted an increased excitatory tone to the spinal cord^6, 7^. The different ion channels and receptors in neurons determine the excitability of sensory neurons, including voltage-gated ion channels and ligand-gated ion channels. Studies have shown that plasticity of voltage-gated ion channels on primary sensory neurons was involved in visceral hypersensitivity induced by diabetes. For example, the function and expression of Kv4.2 were decreased in the colon-specific projecting dorsal root ganglia (DRG) neurons, leading to increased excitability of neurons and colonic hypersensitivity in rats with diabetes^7^. Our previous study also found that changes in Nav1.7 and Nav1.8 expression and function resulted in increased excitability of the colon-specific DRG neurons and diabetic colonic hypersensitivity. Then, what is the role of ligand-gated ion channels in diabetic colonic hypersensitivity?

P2X receptor is a kind of ligand-gated cation channel receptor, and can be passed through by Na^+^, K^+^, Ca^2+^ when it is activated by ATP. It has seven subtypes (P2X1–7)^8^. P2X3 receptors are selectively expressed in primary afferent sensory neurons such as DRGs, trigeminal ganglions and nodose ganglions, especially in medium and small ganglion cells^9, 10^. It was reported that P2X3 receptor was involved in neuropathic pain^11, 12^, inflammatory pain^10^, and visceral hypersensitivity conditions^13^. Our previous studies also found that the expression and function of P2X3 receptors were increased in rats with diabetic neuropathic pain. However, There are few reports about the function of P2X3 receptors in diabetic visceral hypersensitivity. In the present study, we examined the expression of P2X3 receptors and recorded ATP induced-currents in the colon-specific projecting DRG neurons from control and STZ-induced diabetic rats. Our data demonstrated for the first time, to the best of our knowledge, that STZ-induced diabetes increased the expression and function of P2X3 receptors in the colon-specific DRG neurons.

There is growing evidence indicating that oxidative stress may play an important role in the etiology and pathogenesis of diabetes and its complications such as lens cataracts, nephropathy, and neuropathy^14, 15^. Further studies have reported that oxidative stress may change the activities of voltage-gated Na^+^, Ca^2+^ and K^+^ channels in the aging process^16^. Oxidative stress can also affect transient receptor potential subtype ankyrin 1 (TRPA1) channel and mediate oxidant induced itch^17^. Whether oxidative stress is involved in the modulation of P2X3 receptor activities in diabetes is unknown. Alpha-lipoic acid (ALA), a powerful antioxidant, has been increasingly used in several pathological conditions such as diabetic polyneuropathies and inflammation^18^. In the present study, ALA was applied to study the effect of oxidative stress on P2X3 receptors. The results showed that ALA treatment significantly attenuated the colonic visceral hypersensitivity in diabetic rats. ALA treatment also suppressed P2X3 receptor expression, and reduced channel current density.

## Results

### STZ injection induced colonic visceral hypersensitivity

**Figure 1:**
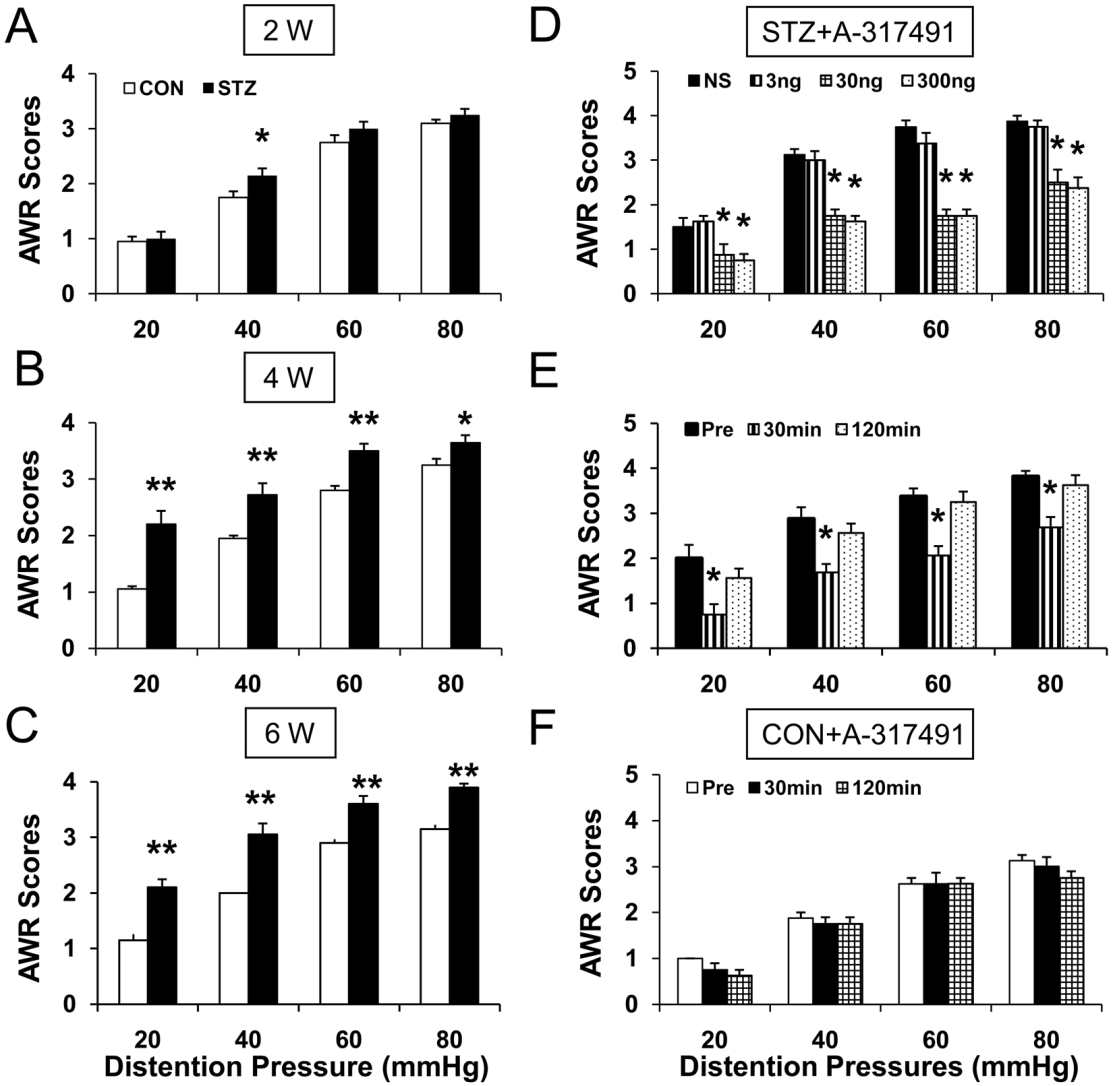
Colonic hypersensitivity in diabetic rats and inhibitory effect of P2X3 receptor antagonist A-317491 on colonic hypersensitivity. (**A**) Abdominal withdrawal reflex (AWR) scores in response to colorectal distention (CRD) from STZ group (n = 10) was significantly higher than CON group (n = 10) at distention pressures of 40 mmHg 2 weeks after vehicle (CON) or STZ (*p < 0.05, compared with CON, Friedman ANOVA). (**B**,**C**) AWR scores in response to CRD from STZ group (n = 10) were significantly higher than control (n = 10) at distention pressures of 20, 40, 60 and 80 mmHg 4 and 6 weeks after STZ or vehicle injection (*p < 0.05, **p < 0.01, compared with CON, Friedman ANOVA). (**D**) A-317491 at doses of 30 nmol/kg (n = 8) and 300 nmol/kg (n = 8) dramatically suppressed AWR scores while NS (n = 8) and A-317491 at 3 nmol/kg (n = 8) had no significant effect on AWR scores (*p < 0.05, compared with NS, Friedman ANOVA). (**E**) 30 min after administration of A-317491 at the dose of 30 nmol/kg resulted in a significant reduction on AWR scores of diabetic rats at 20, 40, 60 and 80 mmHg distention pressures, and the effects cleared away at 120 min after A-317491 treatment (n = 8, *p < 0.05, compared with Pre, Friedman ANOVA). (**F**) A-317491 at the dose of 30 nmol/kg did not produce any effect on AWR in CON rats (n = 6, p > 0.05, compared with Pre, Friedman ANOVA).

After a single intraperitoneal injection of STZ (65 mg/kg body weight), we monitored the blood glucose level and body weight once a week for 6 weeks. In an agreement with our previously published data^19, 20^, majority of rats developed hyperglycemia and displayed polyuria, polydipsia and polyphagia after STZ injection, when compared with CON rats. Blood glucose level elevated 1 week after STZ injection and maintained at a high level for at least another 5 weeks (data not shown). Compared with CON rats, the growth rate of STZ rats was reduced remarkably (data not shown). Abdominal withdrawal reflex (AWR) in response to colorectal distention (CRD) was measured and recorded weekly after STZ or citrate buffer injection. In parallel with elevated blood glucose levels, STZ-induced diabetic rats also developed colonic hypersensitivity. The AWR was increased in 40 mmHg colorectal distention pressure at 2 weeks after STZ injection (Fig. 1A, n = 10 for each group, *p < 0.05, compared with CON, Friedman ANOVA). The AWR was significantly increased in 20, 40, 60, 80 mmHg colorectal distention pressure 4 weeks after STZ injection and lasted for 6 weeks within our observation time period (Fig. 1B and C, n = 10 for each group, *p < 0.05, **p < 0.01, compared with CON, Friedman ANOVA).

### P2XR antagonist A-317491 attenuates diabetic colonic visceral hypersensitivity

P2XR is increasingly thought to be associated with the modulation of pain^10, 11, 13^. To determine whether P2XR is involved in colonic hypersensitivity in STZ-induced diabetic rats, we observed the effect of A-317491, a potent antagonist for P2X3Rs and P2X2/3Rs, on AWR in response to CRD (Fig. 1D and E). A total of 24 diabetic rats were administrated with A-317491 in different doses (3, 30, 300 nmol/kg, i.t.) 4 weeks after STZ injection. AWR were recorded from diabetic rats in responding to CRD 30 mins after A-317491 treatment. Intrathecal injection of A-317491 significantly decreased AWR in diabetic rats (Fig. 1D, n = 8 rats for each group, *p < 0.05, compared with NS, Friedman ANOVA). The maximal inhibitory effects were observed at the dose of 300 nmol/kg for A-317491, and the effects at the dose of 30 nmol/kg were almost the same at the dose of 300 nmol/kg. Since A-317491 at the moderate dose of 30 nmol/kg also produced the maximal effects, the dose of 30 nmol/kg was used to determine the time course of A-317491 effects. As shown in Fig. 1E, the maximal inhibitory effects of A-317491were observed at ~30 min (Fig. 1E, n = 8, *p < 0.05, compared with Pre, Friedman ANOVA), and the effects cleared away at ~2 h after A-317491 treatment. In addition, A-317491 at the dose of 300 nmol/kg did not produce any effect on AWR in CON rats (Fig. 1F, n = 6 rats for each group).

### Function and expression of P2X3Rs were enhanced in DRGs of diabetic rats

**Figure 2:**
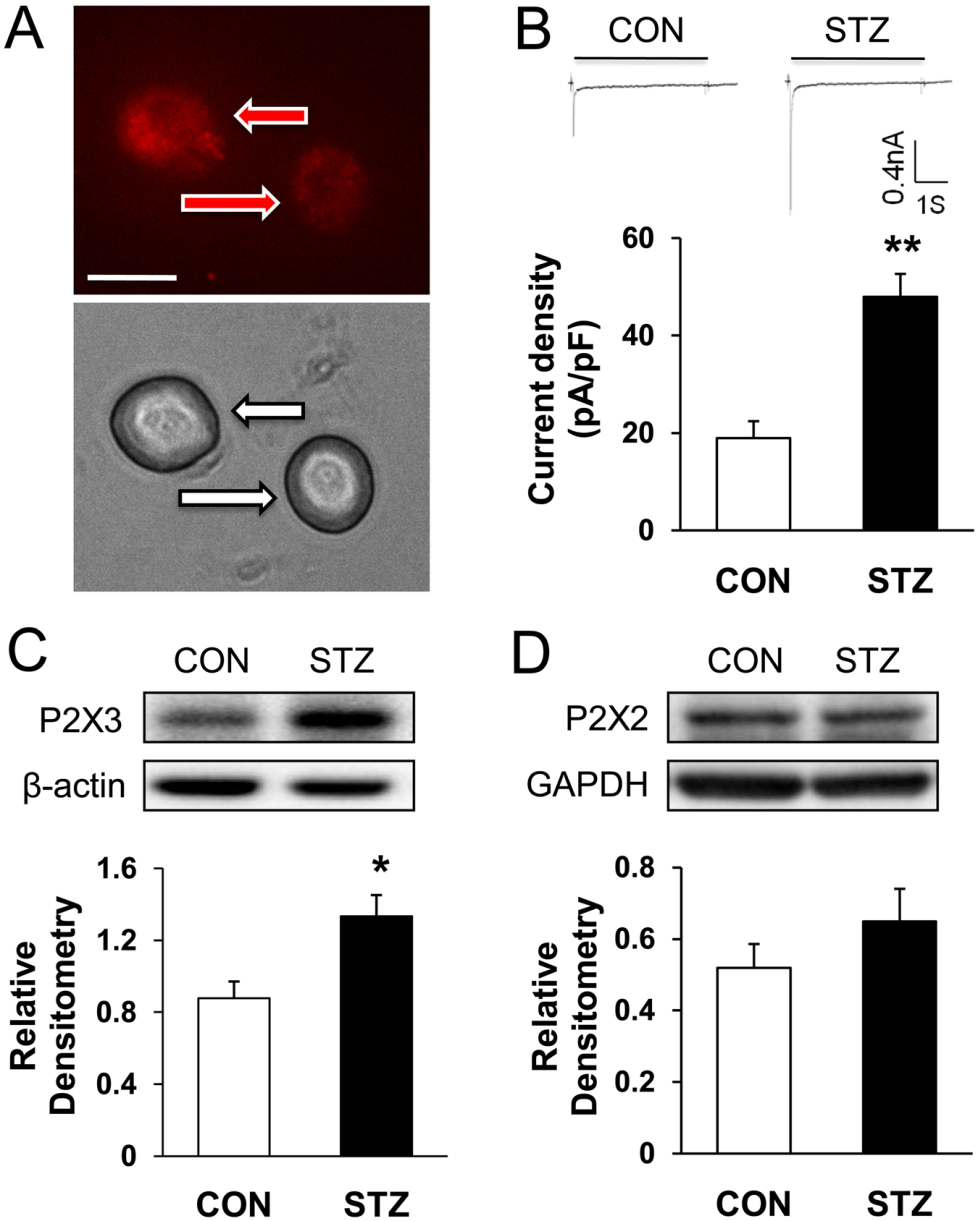
Enhanced ATP current density and upregulated expression of P2X3 Rs in diabetic rats 4 weeks after STZ injection. (**A**) Top: An example of two Dil-labeled DRG neurons (red arrow) innervating colon. Bottom: Phase image of the same DRG neurons labeled by DiI is shown (white arrow). Scale bar = 50 μm. Patch-clamp recordings were performed on DiI-labeled colon-specific neurons. (**B**) Top: An example of ATP evoked inward currents in DiI labeled neurons under voltage clamp conditions. Bottom: Bar graph showing an average of ATP-induced current density of DiI labeled DRG neurons from control and diabetic rats. The average peak current density obtained from control and diabetic rats were 19.0 ± 3.4 pA/pF and 47.9 ± 4.7 pA/pF, respectively (n = 9 cells for each group, **p < 0.01 compared with CON, two sample *t*-test). (**C,D**) Western blots for P2X3R **(C)** and P2X2 R (**D**) of T13-L2 DRGs from CON and STZ-injection rats. Bar graph showed mean density relative to β-actin for P2X3 R and to GAPDH for P2X2 R from CON and STZ rats. STZ injection greatly enhanced expression of P2X3Rs (n = 5 rats for CON, n = 7 rats for STZ, respectively, *p < 0.05, compared with CON, two sample *t*-test), but not significantly altered expression of P2X2 R (n = 5 for each group, p > 0.05, compared with CON, two-sample *t*-test).

To test the hypothesis that function of P2XRs is enhanced in diabetic rats, whole cell patch clamp recording techniques was used to measure the currents evoked by ATP in acutely isolated DRG neurons. Fluorescent dye DiI was used to label colon-innervating DRG neurons (Fig. 2A, top, arrow). At the holding potential of −60 mV, ATP (20 μM) evoked two types of inward currents as described previously^10^: fast inactivating currents and slow inactivating currents. Because the slow inactivating currents was evoked in < 10% of neurons, the present study only included the fast inactivating currents (Fig. 2B, top). The average peak current density in diabetic rats was significantly increased (Fig. 2B, bottom, n = 9 cells for each group, **p < 0.01 compared with CON, two sample t-test). The relative current density was 19.0 ± 3.4 pA/pF (n = 9) and 47.9 ± 4.7 pA/pF (n = 9) in CON and STZ, respectively. In addition, the expressions of both P2X2 R and P2X3 R proteins were measured from T13-L2 DRGs. The expression of P2X3 R was significantly increased in diabetic rats (Fig. 2C, n = 5 rats for CON, n = 7 rats for STZ, respectively, *p < 0.05, compared with CON, two sample *t*-test). In contrast, expression of P2X2 R was not significantly altered 4 weeks after STZ injection (Fig. 2D, n = 5 for each group).

### ALA treatment reduced function and expression of P2X3 Rs in diabetic rats

**Figure 3:**
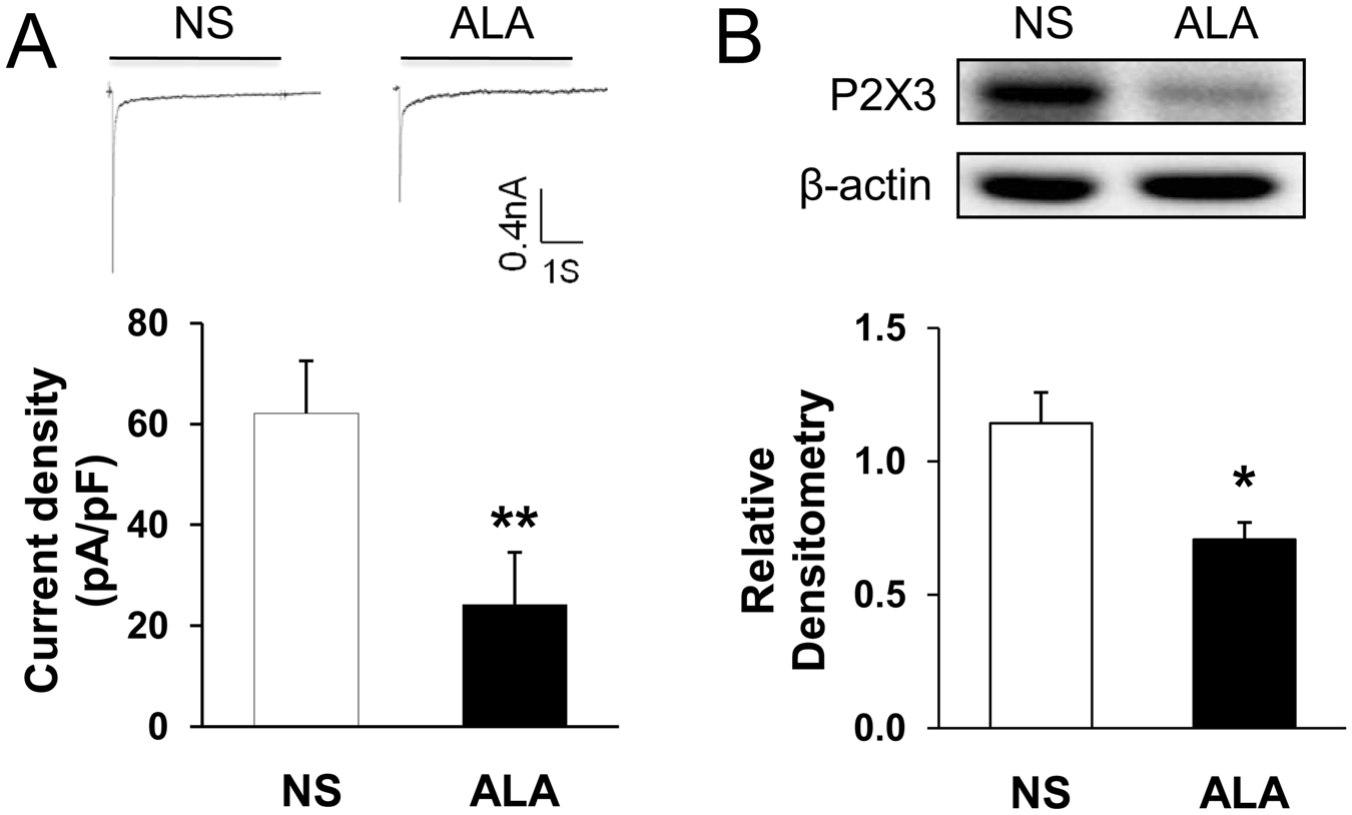
Suppression of ATP current density and P2X3 R expression after ALA treatment in diabetic rats. (**A**) Top: An example of ATP-evoked inward current traces recorded from NS and ALA-treatment rats. Bottom: Bar graph showed a drastical reduction in ATP-evoked current density after ALA treatment (n = 9 cells for each group, **p < 0.01, compared with NS, two-sample *t*-test). (**B**) Western blots for P2X3 Rs of T13-L2 DRGs from NS and ALA-treatment rats. Bar graph showed mean density relative to β-actin for P2X3R. ALA treatment greatly reduced expressions of P2X3Rs (n = 4 for each group, **p < 0.01, compared with NS, two-sample *t*-test).

Alpha-lipoic acid (ALA), a powerful antioxidant, its antioxidant properties have made it a popular treatment in several pathological conditions such as diabetic neuropathy and inflammation^18^. To determine whether ALA has effects on function and expression of P2X3Rs, currents evoked by ATP and P2X3R expression were measured after ALA treatment in diabetic rats. Three weeks after STZ injection, ALA at 60 mg/kg or NS in the same volume was injected intraperitoneally once a day for consecutive 7 days in diabetic rats. ALA treatment significantly suppressed the average peak current density evoked by ATP in diabetic rats (Fig. 3A, **p < 0.01, compared with NS, two-sample *t*-test). The relative current density was 62.1 ± 10.4 (n = 9) and 24.2 ± 3.9 (n = 9) in NS and ALA, respectively. P2X3 R protein expression was also decreased after ALA treatment (Fig. 3B, *p < 0.05, compared with NS, two-sample *t*-test).

### ALA treatment attenuated colonic visceral hypersensitivity in diabetic rats

**Figure 4:**
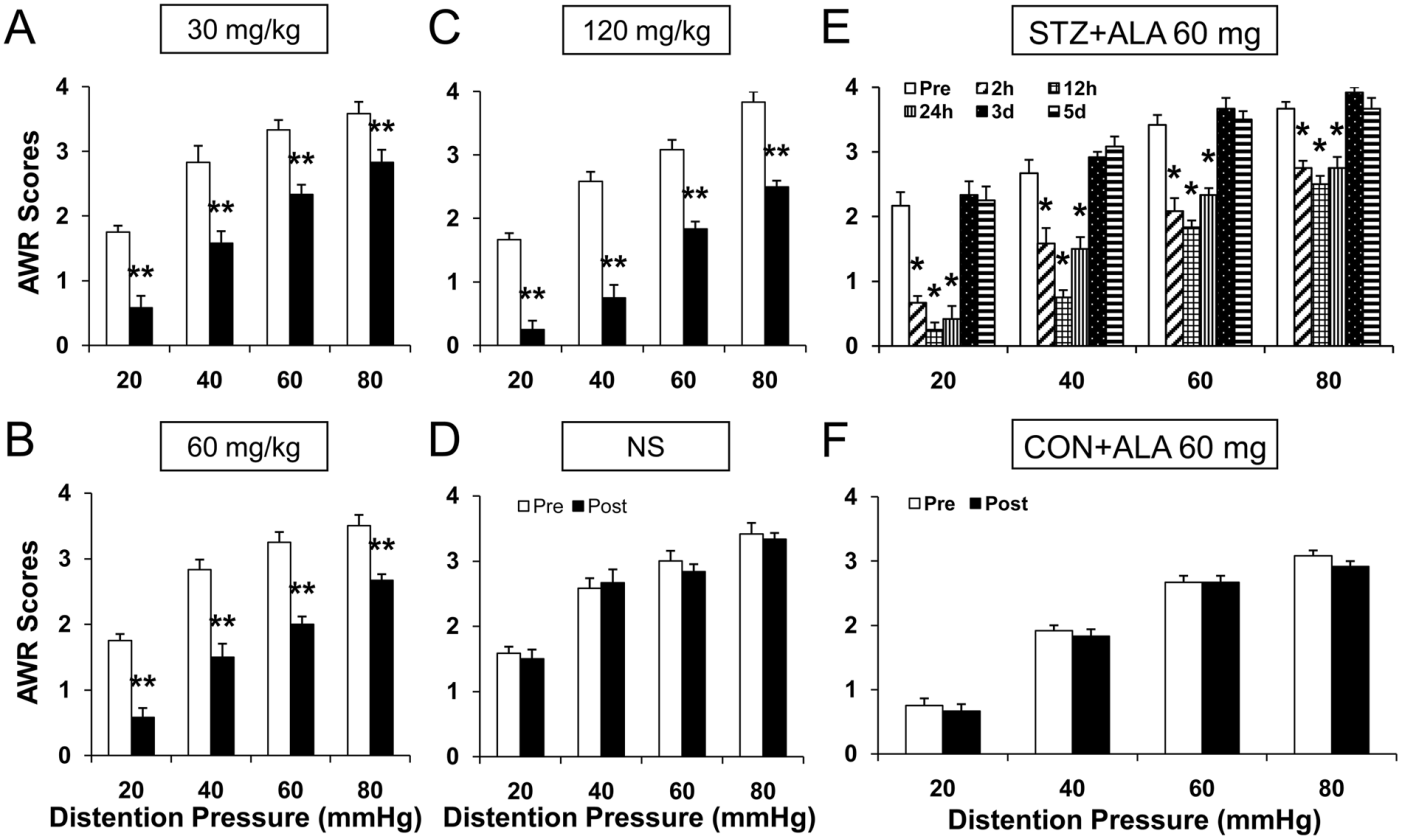
Attenuated colonic hypersensitivity after ALA treatment in diabetic rats. **(A–C)** Treatment with ALA at doses of 30, 60 or 120 mg/kg significantly resulted in a dramatic decrease in the AWR in diabetic rats (n = 6 for each group, **p < 0.01, compared with Pre, Friedman ANOVA). **(D)** NS didn’t change the AWR scores of diabetic rats (n = 6 for each group, p > 0.05, compared with Pre, Friedman ANOVA). **(E)** Time course of ALA induced analgesic effect. ALA at 60 mg/kg significantly increased the AWR scores at 2 hours and lasted for ~24 hours (n = 6 for each group, *p < 0.05, compared with Pre, two-way repeated-measures ANOVA followed by Tukey post hoc test). (**F**) ALA at 60 mg/kg had no significant effect on the AWR scores in age- and sex-matched healthy control rats (n = 6 for each group, p > 0.05, compared with Pre, Friedman ANOVA).

Four weeks after STZ treatment, a total of 18 diabetic rats were administrated with ALA in different doses (30, 60 and 120 mg/kg, i.p.). The AWR to CRD was measured in diabetic rats 30 mins after ALA treatment. ALA at 30, 60 and 120 mg/kg dose all resulted in a dramatic decrease in the AWR scores in diabetic rats (Fig. 4A–C, n = 6 for each group, **p < 0.01, compared with Pre, Friedman ANOVA). NS didn’t alter the AWR scores of diabetic rats (Fig. 4D). The maximal inhibitory effects were observed at the dose of 120 mg/kg for ALA, and the effects at the dose of 60 mg/kg kg were almost the same at the dose of 120 mg/kg. Since ALA at the moderate dose of 60 mg/kg also produced the maximal effects, we next used ALA at 60 mg/kg to determine the time course of ALA effects. As shown in Fig. 4E, the effect of ALA started at ~2 h and lasted for ~24 h. The maximal inhibitory effects of ALA were observed at ~12 h (Fig. 4E, n = 6 for each group, *p < 0.05, compared with Pre, two-way repeated-measures ANOVA followed by Tukey post hoc test), and the effects cleared away at ~3 days after ALA treatment. In addition, ALA at the dose of 60 mg/kg did not change the AWR scores in CON rats (Fig. 4F, n = 6 for each group).

## Discussion

Diabetes leads to many chronic complications including diabetic neuropathy. When the gastrointestinal autonomic nervous system is affected, patients often manifested early satiety, postprandial fullness, bloating, nausea and vomiting, abdominal pain or discomfort, diarrhea or constipation, weight loss, and so on^21, 22^. Because of variations in methodology and patient populations, the prevalence of gastrointestinal symptoms in patients with diabetes is about 5–83% from different reports^2–4^. These gastrointestinal symptoms may be very severe and decrease life quality of patients with diabetes.

Several abnormal gastrointestinal conditions are studied, including disturbed motility and abnormal sensation^23, 24^. Visceral hypersensitivity is one of the important pathological manifestations. In patients, decreased painful threshold of stomach^25^, altered bowel habits, rectal urgency, and diarrhea^26–28^ were observed. In animal models, a variety of stimulations, including balloon distension and electrical impulses, have been used to evaluate gastrointestinal sensation. For example, colonic sensitivity is always evaluated by abdominal withdrawal reflex (AWR) in response to colorectal distention (CRD). Many studies have reported visceral hypersensitivity in diabetic models^7, 20, 24^. The present study also demonstrated that diabetes led to colonic hypersensitivity in rats. The results showed that significant increased AWR scores in response to CRD occurred 2 weeks after injection of STZ and lasted for 6 weeks within our observation time period (Fig. 1A–C).

Colonic hypersensitivity was associated with many factors, including the sensitization of peripheral nociceptive afferents^7, 29, 30^. Changes of ion channel activities in DRG neurons play a significant role in the generation of peripheral sensitization and nociceptive sensation^7, 31, 32^. It was reported that colonic hypersensitivity is associated with decreased opening of K_V_4.2 channels in DRG neurons innervating the colon in rats with diabetes^7^. Our previous study also showed that up-regulated Na_V_1.7 and Na_V_1.8 played important roles in generation of hyperexcitability of DRG neurons and diabetic colonic hypersensitivity^20^. It was well known that ligand-gated channels also play an important role in neuronal sensitization. For example, transient receptor potential vanilloid 1 (TRPV1) receptors were reported to contribute to diabetes-induced alterations in thermal pain sensitivity^33^. More recently, others and we have demonstrated that purinergic P2X receptor antagonists reduced pain behaviors in diabetic mice^34^ and rats^19, 35^. In the present study, we demonstrated that purinergic P2X3 receptors played an important role in the development of diabetic colonic hypersensitivity. The results showed that colonic hypersensitivity was attenuated by a potent P2X3 and P2X2/3 receptor antagonist A-317491 (Fig. 1D–E). In addition, P2X3 R function (Fig. 2A–B) and expression (Fig. 2C) in the colon-specific projecting DRG neurons from rats with diabetes were greatly enhanced 4 weeks after STZ injection. On the contrary, P2X2 receptor expression was not significantly altered (Fig. 2D). Therefore, it is most likely that P2X3 R might be the effective gene in STZ-induced diabetic colonic hypersensitivity.

Then what is the underlying cause of P2X3 R up-regulation in rats with diabetes? A growing body of evidence suggests that oxidative stress may play an important role in the pathogenesis of diabetes and its complications^15, 36^. Oxidative stress is characterized by the depletion of antioxidant defense and excessive production of oxygen free radicals, potentially leading to cell damage and destruction, and ultimately contribute to the development of diabetic complications, including diabetic neuropathy^37–39^. It was reported that diabetic neuropathic pain and hippocampal injury could arise from oxidative stress. Oxidative stress activated the transient receptor potential vanilloid type 1 (TRPV1) and melastatin 2 (TRPM2) channels, leading to the overload of calcium ion (Ca^2+^) entry through the both channels and the ultimate neuronal death^40^. Others also reported that oxidative stress was associated with activated TRPM2 and diabetic neuropathic pain^41^. What’s more, ion channels sensitive to oxidants are not only TRPM2 and TRPV1. P2X2 R can be activated by reactive oxygen species (ROS) through intracellular cys^430^ residue^42^. Overall, oxidative stress induced by diabetes plays an important role on modulating ion channel activity and affecting the excitability of neurons. In addition, there was a report showed that oxidative stress was associated with colonic hypersensitivity^43^. Therefore, we hypothesized that the up-regulation of P2X3 R in colon-specific DRG neurons and diabetic colonic hypersensitivity were induced by oxidative stress. In order to confirm this hypothesis, we applied ALA to treat diabetic colonic hypersensitivity. The results showed that ALA treatment significantly reversed current density induced by ATP in colon-specific DRG neurons (Fig. 3A), down-regulated P2X3 R expression (Fig. 3B), and ultimately attenuated colonic hypersensitivity in rats with diabetes (Fig. 4). The underlying mechanism how oxidative stress regulates the expression and function of P2X3 R remains unknown. It is worth of further study.

In summary, the present study demonstrates that diabetes induced by STZ produces a significant colonic hypersensitivity, which is likely mediated by enhanced function and expression of P2X3 Rs in colon-specific DRG neurons. Treatment with alpha-lipoic acid significantly attenuated colonic visceral hypersensitivity, which might be mediated by down-regulation of P2X3R expression and function. ALA might be a potential therapeutic strategy for colonic visceral hypersensitivity in patients with diabetes.

## Materials and Methods

### Generation of STZ-induced diabetes

All animal experiments in the study were approved by the Institutional Animal Care and Use Committee at the Soochow University, and all methods in the study were performed in accordance with the relevant guidelines and regulations. Adult female Sprague-Dawley (SD) rats (weighing 160~180 g) were housed four per cage in a 12 hour-12 hour light-dark cycle and temperature-controlled (25 ± 1 °C) room. All rats were fed with tap water and standard laboratory chow, *ad libitum*. Streptozotocin (STZ; Sigma Chemicals, St. Louis, MO, USA) was used to induce diabetic models by a single intraperitoneal injection in 65 mg/kg dose, which was freshly dissolved in citrate buffer (10 mmol/L, Na citrate, pH 4.3~4.4), as described previously^35, 44^. The control (CON) rats only received citrate buffer in the same volume. Fasting blood glucose concentration was measured from the tail vein by glucometer (Johnson & Johnson, New Brunswick, NJ, USA) 1 week after STZ injection. Only rats with blood glucose concentration higher than 15.0 mmol/L (270 mg/dL) were further used in the study.

### Measurement of colonic sensitivity

All animals were habituated to the test environment for 1 week before measurement. Colonic sensitivity was measured by recording the response to colorectal distention (CRD) in rats, as described in details previously^20, 45^. Briefly, rats were anesthetized lightly with 1% Brevital (25 mg/kg, i.p.). A flexible latex balloon (6 cm) made from a surgical glove finger and attached to a tygon tubing was inserted 8 cm into the descending colon and rectum via the anus and fixed by taping the tubing to the tail. Rats were placed in small Lucite cubicles and allowed to adapt for 30 min. CRD was performed by rapidly inflating the balloon using a sphygmomanometer. The balloon was inflated to constant pressure 20, 40, 60, and 80 mmHg for 20 s followed by 2 min rest. Abdominal withdrawal reflex (AWR) response to colorectal distention (CRD) was examined and recorded. AWR scores were scored either 0 (normal behavior), 1 (slight head movement without abdominal response), 2 (contraction of abdominal muscles), 3 (lifting of abdominal wall), or 4 (body arching and lifting of pelvic structures). Each measurement was performed twice. All behavioral studies were performed in a blinded manner.

### Cell retrogradely labeling

As described previously^20, 44^, DRG neurons innervating the colon were labeled by fluorescent dye 1, 1′-dioleyl-3, 3, 3′, 3-tetramethylindocarbocyanine methanesulfonate (DiI; Invitrogen, Carlsbad, California), which was injected into the colon wall. Briefly, rats were anesthetized with chloral hydrate (0.36 g/kg, i.p.), and the abdomen was opened and the colon was exposed. DiI (20 mg in 0.5 ml methanol) was injected at 10 sites on the descending colon in a 0.1 μl volume each site. One week later, the abdominal incision healed and STZ or citrate buffer was injected intraperitoneally.

### Acute dissociation of DRG neurons and whole-cell patch clamp recordings

As described previously^10, 44^, bilateral T13-L2 DRGs were dissected out and quickly moved to an ice-cold, oxygenated fresh dissecting solution. Then the DRGs were incubated by collagenase D (2.0–2.2 mg/ml; Roche, Indianapolis, IN) and trypsin (1.5 mg/ml; Sigma, St. Louis, MO) in a 5-ml dissecting solution for 1.5 h at 34.5 °C. After the digestion and washing, a single cell suspension was transferred onto acid-cleaned glass cover slips. 30 min later, whole-cell patch clamp recordings were performed. DiI-labeled neurons were identified under an inverting fluorescence microscope (Olympus IX71, Tokyo, Japan). Small and medium-sized DRG neurons in red were recorded. ATP-evoked currents were recorded by whole-cell patch clamp recordings under voltage clamped mode, as described previously^35^. The external solution contained (mM): NaCl 130, KCl 5, KH_2_PO_4_ 2, CaCl_2_ 2.5, MgCl_2_ 1, HEPES 10, glucose 10 (pH = 7.2, adjusted with NaOH, osmolarity = 295–300 mOsm). The pipette solution contained (mM): potassium gluconate 140, NaCl 10, HEPES 10, glucose 10, BAPTA 5 and CaCl_2_ 1 (pH = 7.25 adjusted with KOH; osmolarity = 295 mOsm). ATP-induced currents were filtered at 2–5 kHz and sampled at 50 or 100 μsec per point.

### Western blotting analysis

Expressions of P2X2 and P2X3 in T13-L2 DRGs from diabetic and CON rats were determined using western blotting analysis, as previously described in details^19, 35^. The primary antibodies including rabbit anti-P2XRs (1:1000, Abcam, USA), rabbit anti-GAPDH (1:1000, Biotechnology Co., CHN), mouse anti-actin (1:1000; Chemicon, Temecula, CA) and the secondary antibodies including anti-rabbit peroxidase-conjugated secondary antibody (1:2000; Santa Cruz Biotechnology, Santa Cruz, CA), anti-mouse horseradish peroxidase-conjugated secondary antibody (1:4000; Chemicon) were used to probe the target proteins.

### Statistical analysis

All data were expressed as means ± SEM. Statistical analysis was conducted using software OriginPro 8 (OriginLab, US) and Matlab (Mathworks, US). Normality was checked for all data before analysis. Significance was determined using Friedman ANOVA, two-way repeated-measures ANOVA followed by Tukey post hoc test and two-sample t-test. Results were considered statistically significant when a p value was less than 0.05.
